# The obesity and inflammatory marker haptoglobin attracts monocytes via interaction with chemokine (C-C motif) receptor 2 (CCR2)

**DOI:** 10.1186/1741-7007-7-87

**Published:** 2009-12-17

**Authors:** Margherita Maffei, Marcella Funicello, Teresa Vottari, Olimpia Gamucci, Mario Costa, Simonetta Lisi, Alessandro Viegi, Osele Ciampi, Giuseppe Bardi, Paolo Vitti, Aldo Pinchera, Ferruccio Santini

**Affiliations:** 1 Dulbecco Telethon Institute, CNR, Area della Ricerca di Pisa, Pisa, Italy; 2 Neurosciences Institute, CNR, Area della Ricerca di Pisa, Pisa, Italy; 3 Scuola Normale Superiore, Pisa, Italy; 4 Department of Psychiatry, Neurobiology, Pharmacology and Biotechnology, University of Pisa, Pisa, Italy; 5 Department of Endocrinology and Kidney, University Hospital of Pisa, Pisa, Italy

## Abstract

**Background:**

Obesity is a chronic low inflammatory state. In the obesity condition the white adipose tissue (WAT) is massively infiltrated with monocytes/macrophages, and the nature of the signals recruiting these inflammatory cells has yet to be fully elucidated. Haptoglobin (Hp) is an inflammatory marker and its expression is induced in the WAT of obese subjects. In an effort to elucidate the biological significance of Hp presence in the WAT and of its upregulation in obesity we formulated the hypothesis that Hp may serve as a macrophage chemoattractant.

**Results:**

We demonstrated by chemotaxis assay that Hp is able to attract chemokine (C-C motif) receptor 2 (CCR2)-transfected pre-B lymphocytes and monocytes in a dose-dependent manner. Moreover, Hp-mediated migration of monocytes is impaired by CCR2-specific inhibition or previous cell exposure to monocyte chemoattractant protein 1 (MCP1) (also known as CCR2 ligand or chemokine (C-C motif) ligand 2 (CCL2)). Downstream effects of Hp/CCR2 interaction were also investigated: flow cytometry proved that monocytes treated with Hp show reduced CCR2 expression on their surface; Hp interaction induces calcium release that is reduced upon pretreatment with CCR2 antagonist; extracellular signal-regulated kinase (ERK)1/2, a signal transducer activated by CCR2, is phosphorylated following Hp treatment and this phosphorylation is reduced when cells are pretreated with a specific CCR2 inhibitor. Consistently, blocking the ERK1/2 pathway with U0126, the selective inhibitor of the ERK upstream mitogen-activated protein (MAP)-ERK kinase (MEK), results in a dramatic reduction (by almost 100%) of the capability of Hp to induce monocyte migration.

**Conclusions:**

Our data show that Hp is a novel monocyte chemoattractant and that its chemotactic potential is mediated, at least in part. by its interaction with CCR2.

## Background

Haptoglobin (Hp) is an acute phase protein synthesized by the liver, and its serum concentrations are elevated during inflammation. Several functions have been attributed to this protein including its ability to bind free hemoglobin, thus preventing oxidative damage, and its capacity to induce angiogenesis [1]. Hp is also expressed by murine and human white adipose tissue (WAT) and, as reported previously, its expression is induced in obesity [2,3]. According to Fain *et al*. [4], Hp is released both by human isolated adipocytes and the adipose tissue matrix, but not by cells of the stromal vascular fraction (SVF). This result is in agreement with the observation of do Nascimento *et al*. [5], who showed that in murine adipose tissue Hp is one of those few inflammatory molecules specifically produced by adipocytes and not present in the SVF. Taken together, these data point to Hp as a novel adipokine as well as a further molecule marking the intersection between obesity and inflammation.

Indeed, the most recent theories [6] describe obesity as a low chronic inflammatory state, and this has been implicated in the development of common medically important complications, including atherosclerosis, hepatic steatosis and insulin resistance [7-9]. Markers of the obesity-induced inflammatory state are the augmented production by adipose tissue, liver and muscle of proinflammatory proteins such as Hp, procoagulant factors, cytokines and chemokines. It is also known that obesity is associated with increased infiltration of macrophages in the WAT, but not in liver and muscle [10]. This accumulation of monocytes/macrophages certainly contributes to the inflammatory-like gene expression pattern displayed by the adipose tissue of the obese, and strong evidence suggests a causal role for macrophages in the onset of insulin resistance in mice [11]. The mechanisms underlying macrophage recruitment are still a matter of investigation, and likely involve increased secretion of chemotactic molecules by the adipocytes. A critical role as a modulator of the influx of monocytes in WAT has been established for the couple ligand/receptor monocyte chemoattractant protein 1 (MCP1; also known as chemokine (C-C motif) receptor 2 (CCR2) ligand or chemokine (C-C motif) ligand 2 (CCL2)) [12,13].

In an effort to further elucidate the biological significance of Hp's presence in the WAT and of its upregulation in obesity we formulated the hypothesis that Hp may serve as a macrophage chemoattractant. We addressed the present issue *in vitro *by assessing the capacity of Hp to attract monocytes (both primary and established cell lines). Our results provide convincing evidence that the starting hypothesis is correct. Further, they suggest that the capacity of Hp to induce macrophage migration is at least partly mediated by its interaction with a chemokine receptor and by the activation of a mitogen-activated protein (MAP) kinase (MAPK) pathway.

## Results

### Haptoglobin chemotaxis studies

**Figure 1:**
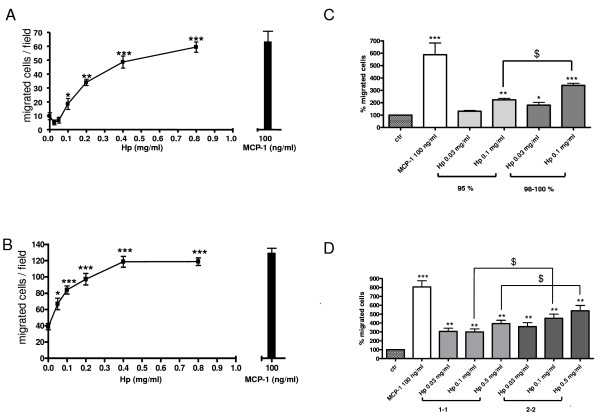
**Effect of haptoglobin (Hp) on U937 monocytes (a, c and d) and human primary monocytes migration (b)**. (a) Chemotaxis was performed on U937 cells for 3 h at the indicated doses of Hp and monocyte chemoattractant protein 1 (MCP1). In the case of the negative control bovine serum albumin (BSA) (1 mg/ml) was used. (b) Chemotaxis was performed on primary monocytes for 90 min. One-way analysis of variance (ANOVA), *P *< 0.0001. Bonferroni post-test versus BSA *P < 0.05, ***P < 0.001. (c, d) Chemotaxis was performed on U937 cells for 3 h at the indicated doses. Migrated cells are expressed as percentage of the average number of migrated cells in the negative control wells (BSA, 1 mg/ml). (c) Bar graph showing the comparison between the chemotactic potential of Hp 95% pure (premade mixture of phenotypes 1-1, 2-1 and 2-2, light gray bars) and that of Hp 98% to 100% pure (homemade mixtures of phenotypes 1-1 and 2-2, dark gray bars). One-way ANOVA, *P *< 0.0001. Bonferroni post-test versus BSA *P < 0.05, **P < 0.01, ***P < 0.001. Bonferroni post-test Hp 95% versus Hp 98% to 100% $*P *< 0.05. (d) Bar graph showing the comparison between chemotactic potential of Hp 1-1 (light gray bars) versus Hp 2-2 (dark gray bars). One-way ANOVA, *P *< 0.0001. Bonferroni post-test versus BSA **P < 0.01, ***P < 0.001. Bonferroni post-test Hp 1-1 versus Hp 2-2 $*P *< 0.05. Data are expressed as means ± standard error of the mean (SEM) of migrated cells for at least three independent experiments.

To our knowledge Hp chemotactic activity has never been previously reported. To assess the capacity of this glycoprotein to attract monocytes/macrophages we performed chemotaxis assays with an established cell line of human monocytes (U937 cells) and with primary monocytes isolated from healthy donors. For both cell types Hp induced a dose-dependent and positive effect on monocyte migration, as shown in the representative curves of Figure 1a, b. MCP1 was used as a positive control. We obtained similar results in five additional experiments, which overall indicated a significant effect of Hp on monocyte migration starting from concentrations of 0.05 and 0.1 mg/ml (approximately 0.435 and 0.87 μM, respectively). This is indicated as an approximate concentration, since in most cases we employed a purchased mixture of the three major human Hp phenotypes, namely 1-1, 2-2 and 2-1 and a precise calculation cannot be performed. For this reason doses employed will be indicated herein with weight/volume measurement units. Hp circulates in human plasma at concentrations ranging from 0.3 to 3 mg/ml (approximately 2.61 to 26.1 μM) [3,14-16] and doses employed throughout the study are within this range or lower. MCP1 was used at concentrations between 10 and 100 ng/ml (1.15 to 11.5 nM), known to induce maximal chemotactic response as reported in the literature [17] and directly assessed in our laboratory (data not shown). The use of Hp in the ng range with human cells would be of scarce physiological significance, given the much higher doses of the protein to which human tissues are normally exposed. To control for possible aspecific effects due to the high amount of the protein employed in case of Hp, experiments were always performed employing similarly high doses of a neutral agent (bovine serum albumin (BSA)) as negative control. BSA did not show chemotactic effects at doses as high as 1 mg/ml (15.1 μM). Results totally overlapping with those shown for U937 undifferentiated monocytes were obtained when U937 cells were induced to differentiate to macrophages (data not shown).

Since in migration assays cells can only move in one direction (that is, towards the filter membrane), the assay must be set up to discriminate between directional and random migration. By following the method established by Heit *et al*. [18] we tested migration across membranes where a chemotactic gradient existed across the membrane (that is, chemoattractant only in the lower well), as well as across membranes where a uniform concentration of chemoattractant was present (that is, chemoattractant in both wells). If the ratio between the number of cells migrating in the gradient versus the number moving in the uniform concentration (termed the chemotactic ratio) is <1 the cells are moving randomly. Chemotactic ratios of >1 suggest that the cells are undergoing chemotaxis. When U937 cells were used we obtained a chemotactic ratio of 1.81 for Hp (tested at 0.5 mg/ml) and of 1.41 for MCP1 (100 ng/ml). This enabled us to establish that what we observed was directional chemotaxis and not chemokinesis.

In the experiments presented in this study we used a purchased mixture of the three Hp phenotypes, namely 1-1, 2-2, and 2-1 (purity 95%). To further confirm our results we performed chemotaxis experiments on U937 monocytes employing the two Hp phenotypes (1-1 and 2-2) at higher purity (98 to 100%) separately or together as a home-made mixture. Data are shown in the bar graphs of Figure 1c, d and indicate that: (1) increasing Hp purity (98% to 100% versus 95%) results in a moderate increase in Hp chemotactic power as demonstrated by the generally increased number of migrated cells toward the 98% pure preparation (Figure 1c), this ruling out the possibility that copurified contaminants (higher in the less purified reagent) other than Hp itself may be responsible for the observed cell migration; (2) the two isoforms slightly differ in that 2-2 at 0.5 and 0.1 mg/ml concentration shows a moderately albeit significantly higher chemotactic potential (Figure 1d). Differences between the use of the 95% pure and 98% to 100% pure reagents were not considered substantial for the main purpose of this study and the experiments described in the following paragraphs were performed using the 95% recombinant Hp.

### Pre-B lymphocytes stably expressing CCR2 are functionally responsive to Hp

The results above indicate that Hp is able to induce chemotaxis. It is well accepted that monocyte and macrophage migration is principally mediated by chemokine-like factors. Indeed, monocytes express abundant levels of active chemokine receptors among which CCR2 has been importantly implicated in macrophage chemoattraction [19] to WAT, where Hp is abundantly expressed and released during obesity. We therefore wanted to evaluate the possibility that Hp might interact with this receptor.

**Figure 2:**
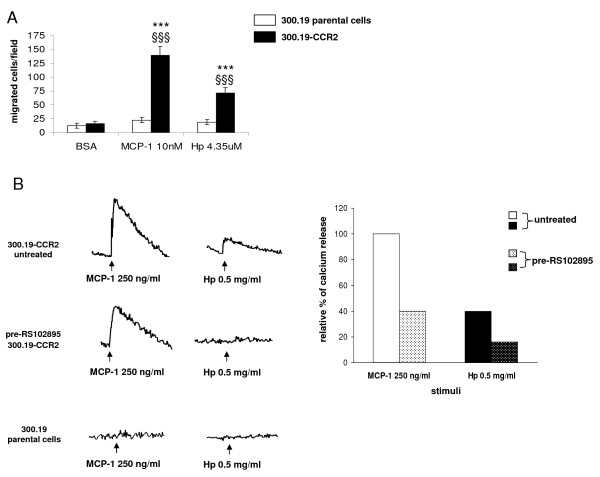
**Effect of haptoglobin (Hp) on cell migration and calcium release in pre-B lymphocytes 300.19 stably expressing chemokine (C-C motif) receptor 2 (CCR2) (300.19-CCR2)**. **(a) **Chemotaxis was performed on 300.19 and 300.19-CCR2 cells for 3 h at the indicated doses of Hp and monocyte chemoattractant protein 1 (MCP1). Bovine serum albumin (BSA) (1 mg/ml) was used as negative control. Two-way analysis of variance (ANOVA), *P *< 0.0001. Bonferroni post-test versus BSA ***P < 0.001, versus 300.19 parental cells §§§*P *< 0.001. **(b) **Left panel, fura-2 acetoxymethyl ester (fura-2 AM) preloaded 300.12-CCR2 cells untreated or pretreated with 5 μM RS102895 were stimulated with 250 ng/ml MCP1 or 0.5 mg/ml Hp, right panel quantification of [Ca^2+^]_i _rise in 300.19-CCR2 cells. The rate of [Ca^2+^]_i _rise (percentage fura-2 saturation/s) induced by MCP1 was set to 100% and the rate after pretreatment was calculated. At the bottom on the left, stimulation of 300.19 parental cells did not result in any appreciable calcium flux. The results are representative of three independent experiments.

To address this issue we first evaluated the chemotactic potential of Hp on murine pre-B cell line 300.19 stably expressing human CCR2 receptor (300.19-CCR2), considered a reliable model to test the specific CCR2 response [20]. Cells transfected with CCR2 migrated towards MCP1 as expected and also positively responded to Hp. 300.19-CCR2 cells did not show any significant migration towards the negative control (BSA 1 mg/ml). Parental cells were neither responsive to MCP1 nor to Hp (Figure 2a).

As calcium flux is one of the most reliable indicators of signaling through chemokine receptors [21], we also evaluated intracellular calcium release in 300.19-CCR2 cells upon MCP1 (250 ng/ml) and Hp (0.5 mg/ml) stimulation. Hp induced an increase of intracellular free calcium in cells preloaded with fura-2 acetoxymethyl ester (fura-2 AM): the amplitude of the signal was approximately 40% of that assessed for MCP1 (Figure 2b). After 300.19-CCR2 cells treatment with the specific CCR2 antagonist RS102895 (5 μM) cells showed a significant decreased responsiveness (less than 60%) to MCP1, while Hp-induced calcium flux was totally abolished (Figure 2b). Hp did not induce any significant calcium flux in parental cells or MCP1.

These data indicate that Hp induces functional responses in pre-B lymphocytes stably expressing CCR2.

### Hp-mediated functional response in monocytes is reduced by CCR2 agonist or antagonist

**Figure 3:**
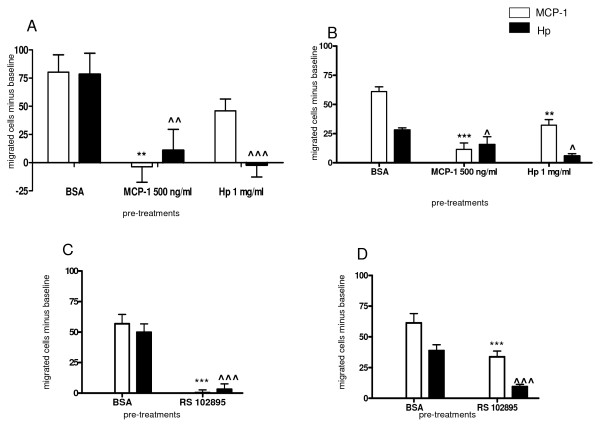
**Monocyte chemoattractant protein 1 (MCP1) and haptoglobin (Hp) show reciprocal interference on the capacity of cells to migrate**. **(a) **Bar graph showing the effect of pretreatment (45 min at 37°C) with MCP1 (500 ng/ml), Hp (1 mg/ml) or bovine serum albumin (BSA) (1 mg/ml) on the capacity of U937 cells to migrate towards MCP1 (100 ng/ml white bars) and Hp (0.5 mg/ml, black bars). For each treatment the number of cells that migrated towards a chemotactically neutral agent (BSA 1 mg/ml) was considered as a baseline and subtracted from the numbers obtained for migration against MCP1 and Hp. Data are expressed as means ± standard error of the mean (SEM) of migrated cells for at least four experiments. Two-way analysis of variance (ANOVA) on the effect of pretreatment, *P *< 0.001. Bonferroni post-test versus pretreatment with BSA **P < 0.01 for migration towards MCP1, ^*P *< 0.01 for migration towards Hp. **(b) **As (a), using human primary monocytes. Two-way ANOVA on the effect of pretreatment, *P *< 0.001. Bonferroni post-test versus pretreatment with BSA ***P 0.001 **P < 0.01 for migration towards MCP1, ^*P *< 0.05 for migration towards Hp. **(c) **Bar graph showing the effect of pretreatment with the chemokine (C-C motif) receptor 2 (CCR2) synthetic antagonist RS102895 (5 μM) or BSA (1 mg/ml) on the capacity of U937 cells to migrate towards MCP1 (100 ng/ml white bars) and Hp (0.5 mg/ml, black bars). For each treatment the number of cells migrated towards a chemotactically neutral agent (BSA 1 mg/ml) was considered as a baseline and subtracted from the numbers obtained for migration against MCP1 and Hp. Data are expressed as means ± SEM of migrated cells for at least four experiments. Two-way ANOVA on the effect of pretreatment, *P *< 0.001. Bonferroni post-test versus pretreatment with BSA ***P < 0.001 for migration towards MCP1, ^^^*P *< 0.001 for migration towards Hp. **(d) **Same as (c) performed with primary monocytes. Data are expressed as means ± SEM of migrated cells for at least four experiments. Two-way ANOVA on the effect of pretreatment, *P *< 0.0001. Bonferroni post-test versus pretreatment with BSA ***P < 0.001 for migration towards MCP1, ^^^*P *< 0.001 for migration towards Hp.

We next wanted to investigate the interaction between Hp and CCR2 by analyzing the extent to which Hp interferes with the specific CCR2 ligand MCP1 in monocytes. We performed chemotaxis assays with U937 human monocytes pretreated for 45 min either with MCP1 (500 ng/ml), the CCR2-specific ligand, Hp (1 mg/ml) or BSA (1 mg/ml), herein used as a neutral agent. Pretreatment with MCP1 resulted in a complete 100% reduction of cells migrated towards MCP1 and an approximate 76% reduction of cells migrated towards Hp. By contrast, pretreatment with Hp completely abolished migration towards Hp itself and caused a 45% reduction in the capacity of U937 cells to migrate towards MCP1 (Figure 3a, bar graph). When higher doses of Hp were employed (2.5 mg/ml) for pretreatment, the effect was further magnified with a 91% reduction in the capacity of U937 cells to migrate towards MCP1. Experiments performed on primary monocytes gave similar results in that pretreatment with MCP1 resulted in a 82% reduction of cells migrated towards MCP1 and 40% reduction of cells migrated towards Hp, whereas Hp pretreatment caused a 47.5% reduction of cells migrated towards MCP1 and 79% reduction of cells migrated towards itself. MCP1 and Hp are then reciprocally capable of interfering with each other in their capacity to attract cells, which is consistent with an interaction with a common receptor.

When U937 cells were incubated for 45 min with the CCR2-specific antagonist RS102895 (5 μM), cell responsiveness to MCP1 was reduced by 100% (as compared with pretreatment with BSA; see above) and a significant reduction of 84.5% was observed in the capacity of cells to migrate towards Hp (0.5 mg/ml) (Figure 3c). After pretreatment with RS102895 (5 μM) human primary monocyte migration towards MCP1 and Hp was also significantly reduced: cells preserved only 46% and 76%, respectively, of their responsiveness to MCP1 and Hp (Figure 3d). Blocking CCR2 therefore has a negative effect on Hp chemotactic activity.

**Figure 4:**
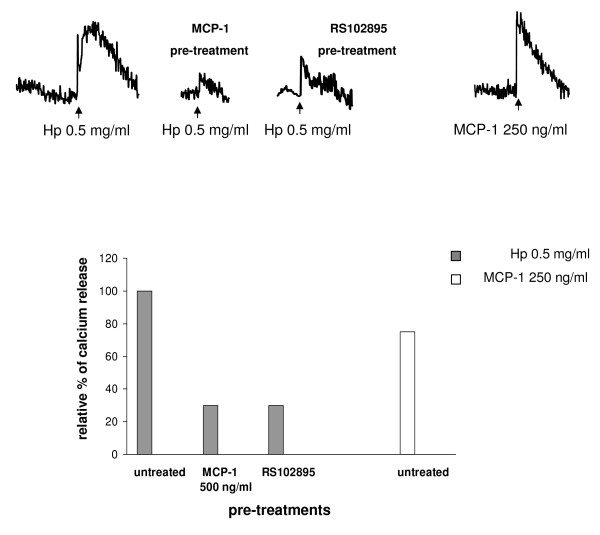
**Effect of haptoglobin (Hp) on calcium release in U937 cells**. Upper panel: fura-2 acetoxymethyl ester (fura-2 AM) preloaded U937 cells untreated or pretreated with 500 ng/ml monocyte chemoattractant protein 1 (MCP1) or 5 μM RS102895 were stimulated with 0.5 mg/ml Hp. Untreated cells were stimulated with 250 ng/ml MCP1 as a positive control. Bottom panel: quantification of [Ca^2+^]_i _rise in U937 cells. The rate of [Ca^2+^]_i _rise (percentage fura-2 AM saturation/s) induced by Hp was set to 100% and the rate after pretreatments was calculated. The result is representative of three independent experiments.

We next evaluated calcium release in U937 cells following Hp stimulation: in this case, and differently to what was observed in 300.19-CCR2 cells, MCP1 and Hp stimulation resulted in similar [Ca^2+^]_i _mobilization (Figure 4). After pretreatment with 500 ng/ml MCP1, cells showed a decreased responsiveness (almost 70%) to 0.5 mg/ml Hp, suggesting that MCP1 interferes with Hp-induced calcium flux. Similar results were obtained after pretreatment with the CCR2 inhibitor RS102895 (65% reduction in calcium release).

Taken together, these data suggest that CCR2 mediates (at least in part) the capability of Hp to attract monocytes and to induce calcium release.

### Hp/CCR2 physical interaction

**Figure 5:**
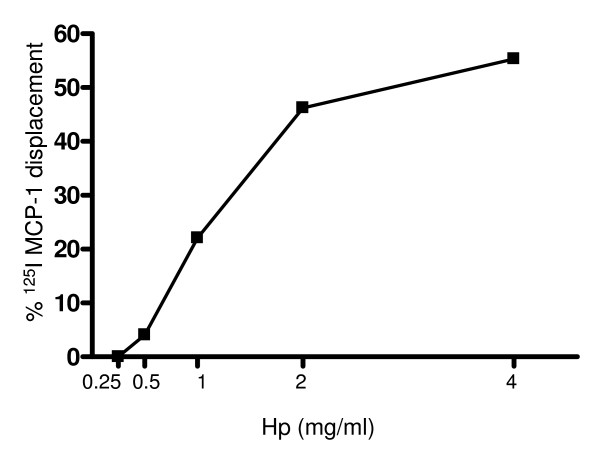
**[^125^I]Monocyte chemoattractant protein 1 (MCP1) displacement from chemokine (C-C motif) receptor 2 (CCR2) by haptoglobin (Hp)**. U937 cells were incubated with the indicated Hp concentrations for 1 h at room temperature and the specific radioactivity was measured by γ counter after free from bound separation. The graph shows one experiment representative of three.

To gain further insights into the type of interaction occurring between Hp and CCR2, we performed binding studies using U937 cells. The curve in Figure 5 shows that Hp is able to displace [^125^I]MCP1 binding to U937 cells in a dose-dependent manner, with a 50% inhibition at a Hp concentration of 2 mg/ml. This suggests that Hp interacts with CCR2 albeit with a low binding affinity.

### Hp induces CCR2 internalization in monocytes

As with other chemokines with agonistic activity, CCR2 activation is followed by internalization [22] and several studies reported CCR2 disappearance from cell surface following exposure to MCP1 [21].

**Figure 6:**
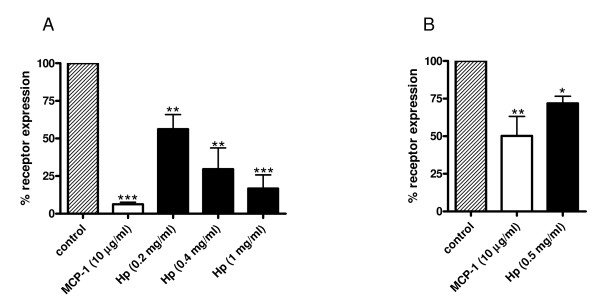
**Haptoglobin (Hp) induces chemokine (C-C motif) receptor 2 (CCR2) internalization**. U937 cells and primary monocytes were incubated for 60 min at 37°C, 5% CO_2 _with increasing concentrations of Hp or monocyte chemoattractant protein 1 (MCP1) as positive control. The relative surface expression of CCR2 was determined by flow cytometry after staining with monoclonal antibodies to the chemokine receptor. **(a) **Bar graph showing the relative mean fluorescence on U937 cells. The dark-striped white bar shows CCR2 expression on U937 cells without treatment. The white bar indicates the percentage of receptor present on the surface after incubation with MCP1 (10 μg/ml) and the black bars indicate the percentage of receptor present on the surface after incubation with Hp at the indicated concentrations. **(b) **Bar graph showing the relative mean fluorescence on primary monocytes. The dark-striped white bar shows CCR2 expression on U937 cells without treatment. The white bar indicates the percentage of receptor present on the surface after incubation with MCP1 (10 μg/ml) and the black bars indicate the percentage of receptor present on the surface after incubation with Hp at 8.7 μM. Data are expressed as means ± standard error of the mean (SEM) of three independent experiments. Student *t *test on the effect of treatment versus control: *P < 0.05; **P < 0.01; ***P < 0.001.

To further prove the potential interaction between CCR2 and Hp, we studied the effect of Hp on CCR2 internalization. MCP1 (herein used as a positive control) and Hp pretreatments induced a dose-dependent disappearance of CCR2 receptor from the surface of U937 cells (Figure 6a) as assessed by flow cytometric analysis.

A similar, albeit less dramatic effect was observed in primary monocytes (Figure 6b). Differences in CCR2 surface expression could partly account for the less pronounced internalization observed in these cells. Indeed, when assessed by flow cytometry primary monocytes displayed on average approximately 50% of the CCR2 surface expression found in U937 cells (data not shown).

We next wanted to rule out the possibility that the observed CCR2 disappearance from cell surface was due to Hp interference with CCR2 binding to its antibody. To this end U937 cells were treated with 0.5 mg/ml Hp or BSA, fixed, permeabilized and stained. When compared to cells similarly treated with Hp but not permeabilized, these samples showed a 50% increase in CCR2 expression (data not shown). This is a further indication that Hp induces CCR2 internalization.

### Hp promotes CCR2 signaling

**Figure 7:**
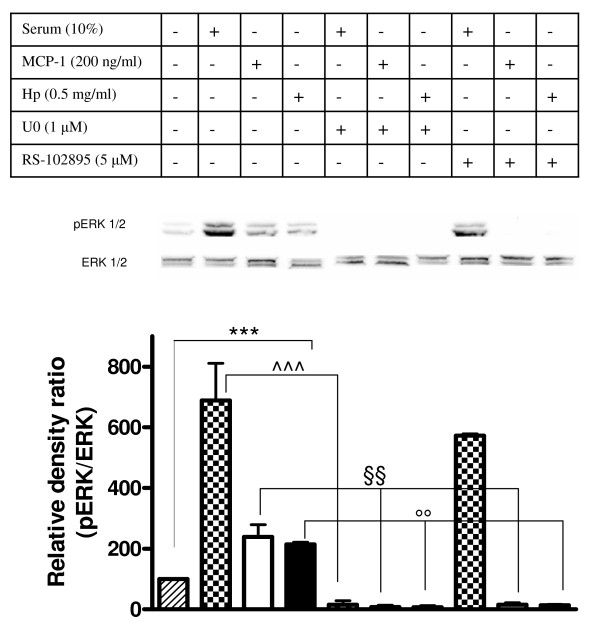
**Haptoglobin (Hp) induces extracellular signal-regulated kinase (ERK) activation in monocytes**. U937 cells were serum starved with 1% serum overnight. Cells were then pretreated or not treated with U0126 (10 min, 1 μM) or RS102895 (30 min, 5 μM) prior to incubation with 10% serum, monocyte chemoattractant protein 1 (MCP1) (200 ng/ml) or Hp (0.5 mg/ml) for 2 min. Harvested cells were lysed and extracted proteins were separated on 12% SDS polyacrylamide gel (50 μg per lane). Activation of ERK1/2 was detected with anti-phospho-ERK antibody. The membrane was stripped and reprobed with anti-ERK1/2 antibody for internal control. In the bottom panel, the bar graph shows the quantification of pERK1/2. Data are expressed as means ± standard error of the mean (SEM) for three experiments. Student *t *test on the effect of serum, MCP1 and Hp versus serum starved cells on ERK1/2 activation. ***P < 0.001. Two-way analysis of variance (ANOVA) on the effect of pretreatment with U0126 or RS102895 on ERK1/2 activation induced by serum, MCP1 and Hp, *P *< 0.0001. Bonferroni post-test. ^^^*P *< 0.001 versus serum stimulated cells, §§*P *< 0.01 versus MCP1 stimulated cells, °°*P *< 0.01 versus Hp stimulated cells.

The MAPK signal transduction pathway is activated in response to the interaction of CCR2 with ligand, and whether this pathway is implicated in the cellular events leading to chemotaxis is a subject of debate [23]. To search for additional evidence that Hp is able to activate CCR2 we assessed the phosphorylation state of extracellular signal-regulated kinase (ERK)1/2 in U937 cells previously starved overnight (1% serum) and subsequently incubated with Hp, with MCP1, or simply stimulated with 10% serum. As shown in the immunoblot and bar graph of Figure 7 there was a significant induction of ERK1/2 phosphorylation in the Hp-treated sample (as compared to starved cells, herein used as control), the intensity of the signal being comparable to that observed for the MCP1-treated samples. When cells were treated with the CCR2 antagonist RS102895 (5 μM) a dramatic decrease in ERK1/2 phosphorylation was observed in the cells treated with Hp and with MCP1, but not in those serum stimulated. Conversely, ERK1/2 phosphorylation was abolished in all types of treatment (Hp, MCP1, 10% serum) when U0126, the selective inhibitor of the ERK upstream kinase MAP-ERK kinase (MEK) [24] was employed.

**Figure 8:**
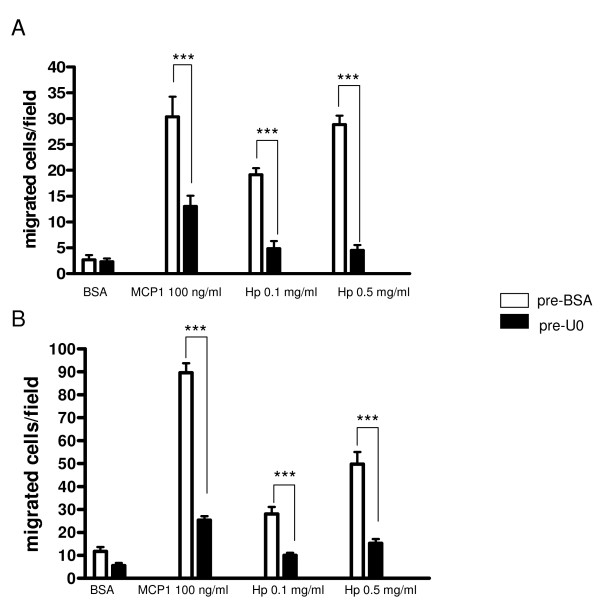
**Haptoglobin (Hp)-mediated chemotaxis is inhibited by blocking extracellular signal-regulated kinase (ERK)1/2 intracellular pathway**. **(a) **Bar graph showing the effect of pretreatment with U0126 (10 min, 1 μM at 37°C, black bars), or bovine serum albumin (BSA) (1 mg/ml, white bars) on the capacity of U937 cells to migrate towards monocyte chemoattractant protein 1 (MCP1) (100 ng/ml) and Hp (0.1 and 0.5 mg/ml). Data are expressed as means ± standard error of the mean (SEM) of migrated cells for at least three experiments. Two-way analysis of variance (ANOVA) *P *< 0.001. Bonferroni post-test versus pretreatment with BSA ***P < 0.001. **(b) **As (a) using human primary monocytes. Two-way ANOVA *P *< 0.001. Bonferroni post-test versus pretreatment with BSA ***P < 0.001.

To further explore the capability of Hp to activate the ERK1/2 pathway, a chemotaxis assay employing Hp and MCP1 as chemotactic agents was performed with U937 cells previously incubated with U0126. The results of this experiment, summarized in the bar graph of Figure 8a, indicate that blocking the ERK1/2 pathway results in a dramatic reduction (by almost 100%) of the capability of Hp and MCP1 to induce cell migration. Totally overlapping results are obtained when primary monocytes are employed (Figure 8b).

These data further strengthen the hypothesis that Hp interacts with CCR2, since activation of ERK1/2 resulting from exposure to Hp is drastically reduced upon pretreatment of cells with a specific CCR2 antagonist. Further, they suggest that an intact ERK1/2 pathway is required for monocyte migration towards Hp and MCP1 to take place.

## Discussion

The results described herein demonstrate that Hp is a novel chemotactic factor and that its capacity to recruit monocytes is mediated (at least in part) by an interaction with the chemokine receptor CCR2. Evidence for this interaction is based on the capacity of Hp to induce CCR2 internalization, the capacity of Hp to bind (albeit with low affinity) CCR2 *in vitro*, Hp induced intracellular calcium flux and Hp activation of the ERK 1/2 pathway. The two latter properties reveal two additional novel roles/functions for Hp. These concepts will be extensively discussed in the following paragraphs.

The *in vitro *evidence reported herein demonstrate that the inflammation/adiposity marker Hp possesses chemotactic potential at doses well within its human physiological concentrations or less [3,15]. Further, our findings highlight differences in the two Hp isoforms 1-1 and 2-2, with the latter being a more potent monocyte chemoattractant. This result should be considered in the light of clinical studies on diabetic patients that indicate an association between the presence of the Hp 2-2 phenotype and a more frequent onset of complications (retinopathy or neuropathy) [25,26] and cardiovascular disease (CVD) [27,28]. These data are extensively reviewed by Nakhoul *et al*. [29] that, among other hypotheses, points to the greater expression of markers of activation in macrophages of type 2-2 (as compared to type 1-1), this implying a higher inflammatory status in these patients. Our *in vitro *findings reinforce this concept by attributing a direct effect of the Hp phenotype on macrophage recruitment. The higher capacity of type 2-2 to recruit macrophages could indeed contribute to enhancing the local inflammatory status, which in turn accelerates the onset of diabetic comorbidities and CVD [30].

The capacity of Hp to recruit monocytes/macrophages also has important implications concerning its role in WAT, where, as we described, its expression and release are importantly induced during obesity [2,3]. Macrophage infiltration in the WAT of obese individuals has been attracting growing attention in the recent years, and has been related to the low chronic inflammatory state that often characterizes obesity status. In particular, the onset of insulin resistance is thought to be determined, at least in part, by the release of inflammatory factors produced by macrophages.

An increasing amount of evidence points to factors actively released by WAT or released into the extracellular spaces when adipocytes undergo cell death and 'explode' [31]. Among these factors particular attention has been dedicated to the chemokine MCP1, which is considered a main player in macrophage recruitment into WAT [32]. Convincing evidence of this role is derived from the increased infiltration of macrophages observed in lean mice overexpressing MCP1 in WAT [33] and, conversely, from the significantly lower content of these cells in obese mice deficient for this factor [12] as compared with controls. However, macrophage content in the WAT of the knockout models was not normalized to the levels observed in lean mice, thus implying the presence of other factors in the modulation of this phenomenon: based on the evidence presented herein, Hp is certainly a good candidate to be considered one such factor.

The MCP1 receptor CCR2 is a G protein-coupled receptor that is predominantly expressed on monocytes and it is thought to be the key receptor mediating their influx into tissues in the context of immune-based inflammation [34]. Recent studies performed *in vivo *in CCR2 knockout (KO) obese animals demonstrated that this role can be extended to the recruitment of monocytes into the adipose tissue of obese subjects [13]. Our work suggests that this receptor may play this role not only by interacting with its high affinity ligand MCP1, but also by interacting with a lower affinity ligand represented by Hp, as reciprocal interference of the two molecules in their capacity to attract monocytes and to induce calcium release indicates.

It has already been proved that chemokines that behave as specific agonists for other chemokine receptors can bind to CCR2 acting as agonists or antagonists [21,35]. Hp could by itself induce migration of mononuclear phagocytes to sites of inflammation even in the absence of MCP1. It is worth noting that one of the major features of inflammatory chemokines is their inducible expression [36], whereas upregulation of Hp is an established chronic condition during obesity. In this context we can see Hp as a 'modulator' of monocyte/macrophage chemoattraction via chemokine receptor 2.

An aspect that differentiates Hp from MCP1 concerns its relative abundance both in plasma, where Hp is 10^8 ^to 10^9 ^times more concentrated than MCP1 (0.3 to 3 mg/ml versus 100 to 400 pg/ml) [15,37] and in the WAT of a lean individual, where Hp is undoubtedly expressed [2,38] whereas MCP1 is almost undetectable [12,33]. These differences should be taken into account when comparing the chemotactic potency of these molecules *in vitro*: if doses in the ng range (10 to 500), such as those required for MCP1, were sufficient for Hp to recruit macrophages, this would result in a very aspecific and potentially harmful effect, taking place in several areas of the body. Consequently, it is conceivable that Hp chemotactic action is associated with the high protein concentrations determined by accumulation of the protein in the WAT of obese individuals.

Tissue distribution also differentiates MCP1 from Hp. MCP1 abundance in WAT is prevalently due (more than 80%) to its expression in the SVF [39] and a recent study by Chung *et al*., [40] indicates that MCP1, along with a number of chemokines and cytokines, is also abundantly expressed in preadipocytes, which play a major role in the inflammatory state of the adipose tissue. Notwithstanding the relevance of MCP1 in macrophage recruitment in WAT, the findings reported above suggest that the MCP1 increase observed in obesity is derived from a number of cell types, including macrophages. As findings by do Nascimento *et al*. clearly indicate [5], Hp gene expression and release are instead almost totally confined to the adipocyte fraction of WAT. Further, our unpublished observations and a proteomic analysis carried out on different stages of adipose conversion revealed that Hp gene expression is linked to the acquisition of the mature adipocyte phenotype [41]. Therefore Hp is one of those few inflammatory molecules specifically expressed by adipocytes within WAT. In summary, these observations suggest that Hp plays a role in monocyte/macrophage recruitment to WAT in conditions slightly different than those typical of MCP1. Those conditions could be part of the switch that, according to Lumeng *et al*. takes place in the WAT during obesity [42] when alternatively activated macrophages, producing anti-inflammatory molecules, are replaced by classically activated macrophages producing inflammatory molecules.

We can therefore speculate that Hp participates mainly in the first part of the process, when adipocytes undergoing the initial effects of weight gain, start producing an increased concentration of molecules, including Hp, that activate the recruitment of other CCR2^+ ^monocytes or that, likely, induce changes in the expression profile of the resident macrophages, which in turn recruit other monocytes (for instance by overexpressing MCP1). Our hypothesis of Hp as a modulator for monocyte/macrophage attraction to WAT does not underestimate the major role played by MCP1 or the other chemokines binding CCR2. In fact, we cannot exclude the possibility that Hp-induced functional responses observed in monocytes are not exclusively due to Hp/CCR2 interaction, but are also due to the action of the glycoprotein on other chemokine receptors. In this regard it is interesting to observe the different potency shown by Hp in inducing calcium release in lymphocytes stably expressing CCR2 and in monocytes. If for the former the Hp-induced [Ca^2+^]_i _signal is less than half of that induced by MCP1, in the case of monocytes calcium mobilization is equally induced by the two factors. This may suggest the existence of Hp-responsive receptors, other than CCR2, expressed by monocytes and not by pre-B lymphocytes.

This study also demonstrates for the first time that Hp is able to induce calcium release and to activate ERK1/2 MAP kinase. This introduces remarkable additions to the functions and properties classically attributed to Hp. As a matter of fact this glycoprotein, so far considered a player in actions mostly occurring in the extracellular environment (hemoglobin transportation, antioxidant capacities), seems to be capable of interfering with the cascade of events activated by calcium signaling among which an intracellular pathway playing a pivotal role in several physiological and pathological conditions. Indeed, cytosolic alterations of calcium ion concentrations are an integral part of signal transduction [43] and reported evidence indicates that in monocytes calcium is a determinant second messenger in the induction of the inflammatory response orchestrated by nuclear factor (NF)κB [44]. This implies that the elevated Hp levels observed in the WAT of obese subjects [2] may be relevant in determining an increase in the production of inflammatory molecules (cytokines, chemokines) in resident or in newly recruited monocytes.

The role of MAPK pathways in monocytes migration has been previously investigated. As reported by Ashida and collaborators [23] in the established THP1 cell line of monocytes, ERK1/2 is responsible for integrin activation but not for chemotaxis, which is under the control of Rho kinase and p38 MAPK. Conversely, according to our data on U937 cells ERK1/2 is indeed implicated both in MCP1-dependent and Hp-dependent chemotaxis.

## Conclusions

This study demonstrates for the first time that Hp is able to recruit monocytes (macrophages) by interacting with the chemokine receptor CCR2. This discloses a novel function for this molecule, which is upregulated in the WAT of obese subjects and which could participate to the massive infiltration of monocytes observed in obesity. Further, we have shown that by pharmacologically inhibiting CCR2 we could block cell migration to Hp, further strengthening the notion that drugs for the obesity-induced inflammatory state could be developed by acting on this receptor. In this regard pharmacological inhibition of the ERK1/2 CCR2 downstream pathway should also be considered as a possible therapeutical intervention to prevent macrophage infiltration into WAT.

## Methods

### Cell culture

Human U937 monocytic cells were purchased from American Type Culture Collection (ATCC Manassas, VA, USA). Cells were maintained as a monocytic cell suspension in T-75 flasks containing RPMI 1640 medium supplemented with 10% fetal bovine serum and antibiotics at 37°C in 5% CO_2_, and cultures were split every 3 to 5 days. The murine pre-B cell line 300.19 stably transfected with human CCR2 receptor was a kind gift of M Uguccioni, IRB Bellinzona, Bellinzona, Switzerland [21].

300.19 cells were cultured in RPMI 1640 supplemented with 10% fetal calf serum (FCS), 1% non-essential amino acids, 1 mM sodium pyruvate, 0.05 mM β-mercaptoethanol, 50 U/ml penicillin, 50 mg/ml streptomycin, 10 mM 4-(2-hydroxyethyl)-1-piperazineethanesulfonic acid (HEPES) and 2 mM glutamine at 37°C in 5% CO_2_. Cells were split every 2 to 3 days and positive clones were selected in the presence of 1.5 μg/ml puromycin (Sigma, St Louis, MO, USA).

### Isolation of monocytes

Peripheral blood mononuclear cells (PBMCs) were isolated from Buffy coats from healthy donor male subjects (aged 20 to 40 years old) obtained from the Blood Transfusion Center of the Cisanello University Hospital (Pisa, Italy). Blood was diluted 1:4 with a solution containing phosphate-buffered saline (PBS) pH 7.2, 0.5% BSA and 2 mM ethylenediaminetetra-acetic acid (EDTA) and then 35 ml of this solution were carefully layered over 15 ml Ficoll-Paque (density = 1.077 g/ml) in a 50 ml conical tube and centrifuged at 400 *g *for 30 min. The interphase cells (PBMCs) were transferred to a new 50 ml conical tube filled with PBS/EDTA and centrifuged at 300 *g *for 10 min. Then, the pellet was washed for removal of platelets by spinning at 200 *g *for 10 min. Monocytes were isolated from PBMCs by magnetic bead separation using Human monocyte isolation kit II (Miltenyi Biotec, Bergisch Gladbach, Germany) resuspended in complete RPMI with 10% FBS and antibiotics, and cultured overnight prior to use.

### Chemotaxis assays

Chemotaxis assays were performed in 48-well Boyden microchambers (AP48, Neuro Probe, Cabin John, MD, USA). In the bottom wells of the chamber we added 30 μl of a serum free 0.1% BSA RPMI solution containing one of the following peptides: human Hp (Sigma, catalog no.s H3536, H0138 and H9762 respectively for the 95% pure mixture, and for the 98% pure 1-1 and 2-2 phenotypes), human MCP1 (Peprotech, Rocky Hill NJ, catalog no. 300-04). BSA at a concentration of 1 mg/ml was used as a negative control. An uncoated 10-μm thick polyvinylpyrrolidone-free polycarbonate filter with a pore size of 5 μm was placed over the samples (Neuro Probe, Gaithersburg, MD). The silicon gasket and the upper piece of the chamber were applied, and 50 μl of the cell suspension (1 × 10^5 ^to 2.5 × 10^5^/50 μl) was placed into the upper wells. Cells were in some cases (referred to as pretreatments) incubated for 45 min at 37°C with MCP1, Hp or BSA or the CCR2-specific antagonist RS102895 (see Results section and Figure legends for concentrations). Following the treatment cells were pelleted, washed and finally resuspended in a suitable volume to perform chemotaxis.

The chamber was incubated in a humidified 5% CO_2 _atmosphere for 1.5 to 3 h at 37°C, and non-migrated cells were gently wiped away from the upper surface of the filter. The filter was immersed for 30 s in a methanol-based fixative and stained with Diff-Quick (Medion Diagnostics AG, CH-3186, Düdingen, Switzerland) and then mounted on a glass slide. Cells that had completely migrated through the filter were counted in 10 random high-power fields (HPF; original magnification × 400) under light microscopy.

### Intracellular calcium measurements

U937 and 300.19 cells were loaded with 3 μM fura-2 AM (Molecular Probes, Invitrogen, San Diego CA) in PBS containing 0.5% BSA for 30 min at room temperature in the dark. After being washed in indicator free medium, cells were resuspended at a density of 1 × 10^6 ^cells/ml in PBS/0.5% BSA containing 1 mM CaCl_2_. After a further incubation of 30 min to allow complete de-esterification of intracellular fura-2 ester cells were ready to be analyzed. Pretreatments with MCP1 (500 ng/ml) and RS102895 (5 μM) were performed for 30 min at room temperature during the indicator de-esterification. The cell suspension (800 μl/sample) was then transferred into glass cuvettes. Cells were stimulated with the appropriate chemokine (250 ng/ml nM MCP1 or 0.5 mg/ml Hp) and real-time data were recovered using a fluorometer (LS55; PerkinElmer, Watham MA). Data were analyzed using FL-Winlab Software (PerkinElmer) and expressed as the relative ratio of fluorescence emitted at 510 nm after sequential stimulation at 340 and 380 nm (excitation wavelengths).

### Radioligand binding studies (whole-cell binding)

Binding studies were performed according to Sarau *et al*. [45]. Briefly U937 cells (5 × 10^5^), resuspended in RPMI with 0.1% BSA and 25 mM HEPES (pH 7.4) (reaction buffer), were incubated with [^125^I]MCP1 (4 ng/ml) in the absence or presence of unlabeled Hp (0.25 to 2 mg/ml) or an excess amount of MCP1 (1 μg/ml) for 1 h at room temperature in Eppendorf microcentrifuge tubes (final reaction volume 100 μl). The binding reaction was terminated by placing the incubation mixture over a 10% sucrose cushion (375 μl) and centrifuging at 14,000 rpm for 2 to 3 min to separate bound from free ligand. The resultant supernatant fraction was discarded, and the amount of the radioactivity associated with the pellet was determined by γ scintillation spectrometry. The percentage of [^125^I]MCP1 displacement from the receptor was calculated using T - sample_i_/T - NS, where T is the total radioactivity measured in the absence of Hp or MCP1, sample_i _is the radioactivity measured when a given concentration of Hp was added and NS (not specific) is the radioactivity measured in the presence of an excess amount of MCP1.

### CCR2 internalization

Incubations of U937 cells or primary monocytes with the different ligands have been performed for 60 min at 37°C, 5% CO_2 _in RPMI medium supplemented with 1% BSA. Internalization of CCR2 was followed by flow cytometry using phycoerythrin-conjugated mouse monoclonal anti-human (25 mg/ml; R&D Systems, **Minneapolis MN**) resuspended in FACS buffer (PBS/1% BSA). Isotype-matched control IgG was used for control staining. Cell associated fluorescence was analyzed by flow cytometry (FACScan, Becton Dickinson, Mountain View, CA, USA).

CCR2 internalization was also evaluated in fixed and permeabilized cells. Briefly, before staining, cells were incubated on ice in 1% paraformaldehyde for 2 min, washed and then permeabilized with 0.15% saponin for 30 min on ice.

### ERK phosphorylation

U937 cells were aliquoted into a Petri dish at 5 × 10^6 ^cells/sample in 1.0 ml of CCR2 binding buffer and prewarmed to 37°C for 10 min. Compound was added for 5 min before stimulation. The samples were stimulated with mCCL2 (30 nM; R&D Systems) for 1 min. The cells were quickly pelleted, the supernatant was removed, and 100 μl of ice-cold lysis buffer containing 50 mM Tris pH 7.4, 150 mM NaCl, 0.25% Na-deoxycolate, 1% nonyl phenoxylpolyethoxylethanol (NP40), protease inhibitor cocktail (Roche, Mannheim Germany, catalog no. 11836153001), phosphatase inhibitor cocktail (1 and 2, Sigma catalog no.s P-2850 and P-5726) was added. After 10 min on ice, the samples were microfuged at 13,000 rpm for 10 min at 4°C, and the supernatants were collected. For western analysis, 15 μl of 2 × Laemmli sample buffer was added to 15 μl of cell extract, and the samples were boiled for 5 min and loaded onto 12% Tris-glycine gels (Bio-Rad, Hercules CA). Following electrophoresis and transfer onto poly(vinylidene difluoride) membrane, the membranes were probed with either a rabbit polyclonal anti-phospho-ERK antibody or rabbit polyclonal anti-ERK to detect total ERK protein (1/1,000 dilution; Cell Signaling Technology, Danvers MA) followed by a HRP-conjugated goat anti-rabbit IgG antibody (1/2,000; Cell Signaling Technology). After washing the blots in PBS + 0.1% Tween 20, the blots were developed with the enhanced chemiluminescence detection system (Bio-Rad). The same membranes were stripped and reprobed with anti-ERK2 for normalization. Signals were acquired by Molecular Imager Chemidoc XRS System (Bio-Rad) and their intensity was quantified by using Quantity One software (Bio-Rad).

### Statistics

All values are expressed as means ± standard error of the mean (SEM) for at least three independent experiments. Pairwise comparisons (for example, CCR2 expression: on cells preincubated with BSA versus cells preincubated with Hp) were assessed by two-tailed Student *t *test. When more than two groups were analyzed, two-way or one-way analysis of variance (ANOVA) followed by Bonferroni post-test for selected comparisons (for example, pretreatment with BSA versus pretreatment with MCP1) was used.

## Authors' contributions

MM conceived and designed the study, supervised the experiments and wrote the paper; MF performed and supervised chemotaxis experiments, analyzed data and prepared figures; TV performed ERK activation studies, analyzed data, performed binding studies and prepared figures; OG performed chemotaxis experiments, prepared primary monocytes and participated in flow cytometry experiments; AV, OG and SL performed calcium experiments; MC supervised ERK activation studies and performed some of the experiments; SL performed flow cytometry experiments and analyzed data; OC performed binding studies; GB designed the study, supervised and contributed to flow cytometry experiments and prepared figures; PV and AP contributed to materials/reagents/analysis tolls and participated in paper writing; FS conceived and supervised binding studies and participated in paper writing. All authors read and approved the final manuscript.

## References

[B1] de Kleijn DP Smeets MB Kemmeren PP Lim SK Van Middelaar BJ Velema E Schoneveld A Pasterkamp G Borst C Acute-phase protein haptoglobin is a cell migration factor involved in arterial restructuring FASEB J 2002 16 1123 1125 12039846 10.1096/fj.02-0019fje

[B2] Chiellini C Bertacca A Novelli SE Görgün CZ Ciccarone A Giordano A Xu H Soukas A Costa M Gandini D Dimitri R Bottone P Cecchetti P Pardini E Perego L Navalesi R Folli F Benzi L Cinti S Friedman JM Hotamisligil GS Maffei M Obesity modulates the expression of haptoglobin in the white adipose tissue via TNFalpha J Cell Physiol 2002 190 251 258 10.1002/jcp.10061 11807829

[B3] Chiellini C Santini F Marsili A Berti P Bertacca A Pelosini C Scartabelli G Pardini E López-Soriano J Centoni R Ciccarone AM Benzi L Vitti P Del Prato S Pinchera A Maffei M Serum haptoglobin: a novel marker of adiposity in humans J Clin Endocrinol Metab 2004 89 2678 2683 10.1210/jc.2003-031965 15181041

[B4] Fain JN Madan AK Hiler ML Cheema P Bahouth SW Comparison of the release of adipokines by adipose tissue, adipose tissue matrix, and adipocytes from visceral and subcutaneous abdominal adipose tissues of obese humans Endocrinology 2004 145 2273 2282 10.1210/en.2003-1336 14726444

[B5] do Nascimento CO Hunter L Trayhurn P Regulation of haptoglobin gene expression in 3T3-L1 adipocytes by cytokines, catecholamines, and PPARgamma Biochem Biophys Res Commun 2004 313 702 708 10.1016/j.bbrc.2003.12.008 14697247

[B6] Ferrante AW Jr Obesity-induced inflammation: a metabolic dialogue in the language of inflammation J Intern Med 2007 262 408 414 10.1111/j.1365-2796.2007.01852.x 17875176

[B7] Berg AH Scherer PE Adipose tissue, inflammation, and cardiovascular disease Circ Res 2005 96 939 949 10.1161/01.RES.0000163635.62927.34 15890981

[B8] Browning JD Horton JD Molecular mediators of hepatic steatosis and liver injury J Clin Invest 2004 114 147 152 15254578 10.1172/JCI22422 PMC449757

[B9] Lazar MA How obesity causes diabetes: not a tall tale Science 2005 307 373 375 10.1126/science.1104342 15662001

[B10] Weisberg SP McCann D Desai M Rosenbaum M Leibel RL Ferrante AW Jr Obesity is associated with macrophage accumulation in adipose tissue J Clin Invest 2003 112 1796 1808 14679176 10.1172/JCI19246 PMC296995

[B11] Arkan MC Hevener AL Greten FR Maeda S Li ZW Long JM Wynshaw-Boris A Poli G Olefsky J Karin M IKK-beta links inflammation to obesity-induced insulin resistance Nat Med 2005 11 191 198 10.1038/nm1185 15685170

[B12] Kanda H Tateya S Tamori Y Kotani K Hiasa K Kitazawa R Kitazawa S Miyachi H Maeda S Egashira K Kasuga M MCP-1 contributes to macrophage infiltration into adipose tissue, insulin resistance, and hepatic steatosis in obesity J Clin Invest 2006 116 1494 1505 10.1172/JCI26498 16691291 PMC1459069

[B13] Weisberg SP Hunter D Huber R Lemieux J Slaymaker S Vaddi K Charo I Leibel RL Ferrante AW Jr CCR2 modulates inflammatory and metabolic effects of high-fat feeding J Clin Invest 2006 116 115 124 10.1172/JCI24335 16341265 PMC1307559

[B14] Delanghe JR Langlois MR Hemopexin: a review of biological aspects and the role in laboratory medicine Clin Chim Acta 2001 312 13 23 10.1016/S0009-8981(01)00586-1 11580905

[B15] Kormoczi GF Saemann MD Buchta C Peck-Radosavljevic M Mayr WR Schwartz DW Dunkler D Spitzauer S Panzer S Influence of clinical factors on the haemolysis marker haptoglobin Eur J Clin Invest 2006 36 202 209 10.1111/j.1365-2362.2006.01617.x 16506966

[B16] Jahoor F Gazzard B Phillips G Sharpstone D Delrosario M Frazer ME Heird W Smith R Jackson A The acute-phase protein response to human immunodeficiency virus infection in human subjects Am J Physiol 1999 276 E1092 1098 10362622 10.1152/ajpendo.1999.276.6.E1092

[B17] Uguccioni M D'Apuzzo M Loetscher M Dewald B Baggiolini M Actions of the chemotactic cytokines MCP-1, MCP-2, MCP-3, RANTES, MIP-1 alpha and MIP-1 beta on human monocytes Eur J Immunol 1995 25 64 68 10.1002/eji.1830250113 7531149

[B18] Heit B Colarusso P Kubes P Fundamentally different roles for LFA-1, Mac-1 and alpha4-integrin in neutrophil chemotaxis J Cell Sci 2005 118 5205 5220 10.1242/jcs.02632 16249234

[B19] Rollins BJ Walz A Baggiolini M Recombinant human MCP-1/JE induces chemotaxis, calcium flux, and the respiratory burst in human monocytes Blood 1991 78 1112 1116 1868242

[B20] Arai H Tsou CL Charo IF Chemotaxis in a lymphocyte cell line transfected with C-C chemokine receptor 2B: evidence that directed migration is mediated by betagamma dimers released by activation of Gα i-coupled receptors Proc Natl Acad Sci USA 1997 94 14495 14499 10.1073/pnas.94.26.14495 9405641 PMC25033

[B21] Ogilvie P Bardi G Clark-Lewis I Baggiolini M Uguccioni M Eotaxin is a natural antagonist for CCR2 and an agonist for CCR5 Blood 2001 97 1920 1924 10.1182/blood.V97.7.1920 11264152

[B22] Mack M Cihak J Simonis C Luckow B Proudfoot AE Plachy J Bruhl H Frink M Anders HJ Vielhauer V Pfirstinger J Stangassinger M Schlondorff D Expression and characterization of the chemokine receptors CCR2 and CCR5 in mice J Immunol 2001 166 4697 4704 11254730 10.4049/jimmunol.166.7.4697

[B23] Ashida N Arai H Yamasaki M Kita T Distinct signaling pathways for MCP-1-dependent integrin activation and chemotaxis J Biol Chem 2001 276 16555 16560 10.1074/jbc.M009068200 11278464

[B24] Costa M Marchi M Cardarelli F Roy A Beltram F Maffei L Ratto GM Dynamic regulation of ERK2 nuclear translocation and mobility in living cells J Cell Sci 2006 119 4952 4963 10.1242/jcs.03272 17105770

[B25] Levy AP Roguin A Hochberg I Herer P Marsh S Nakhoul FM Skorecki K Haptoglobin phenotype and vascular complications in patients with diabetes N Engl J Med 2000 343 969 970 10.1056/NEJM200009283431313 11012324

[B26] Nakhoul FM Marsh S Hochberg I Leibu R Miller BP Levy AP Haptoglobin genotype as a risk factor for diabetic retinopathy JAMA 2000 284 1244 1245 10.1001/jama.284.10.1244-a 10979109

[B27] Suleiman M Kapeliovich MR Roguin A Aronson D Meisel SR Shochat M Reisner SA Hammerman H Lotan R Levy NS Levy AP Haptoglobin type and 30-day mortality in diabetic individuals presenting with acute myocardial infarction Diabetes Care 2003 26 2699 2700 10.2337/diacare.26.9.2699 12941748

[B28] Asleh R Levy AP *In vivo *and *in vitro *studies establishing haptoglobin as a major susceptibility gene for diabetic vascular disease Vasc Health Risk Manag 2005 1 19 28 10.2147/vhrm.1.1.19.58930 17319095 PMC1993923

[B29] Nakhoul FM Miller-Lotan R Awaad H Asleh R Levy AP Hypothesis - haptoglobin genotype and diabetic nephropathy Nat Clin Pract Nephrol 2007 3 339 344 10.1038/ncpneph0467 17525716

[B30] Langlois MR Delanghe JR Biological and clinical significance of haptoglobin polymorphism in humans Clin Chem 1996 42 1589 1600 8855140

[B31] Cinti S Mitchell G Barbatelli G Murano I Ceresi E Faloia E Wang S Fortier M Greenberg AS Obin MS Adipocyte death defines macrophage localization and function in adipose tissue of obese mice and humans J Lipid Res 2005 46 2347 2355 10.1194/jlr.M500294-JLR200 16150820

[B32] Shoelson SE Lee J Goldfine AB Inflammation and insulin resistance J Clin Invest 2006 116 1793 1801 10.1172/JCI29069 16823477 PMC1483173

[B33] Kamei N Tobe K Suzuki R Ohsugi M Watanabe T Kubota N Ohtsuka-Kowatari N Kumagai K Sakamoto K Kobayashi M Yamauchi T Ueki K Oishi Y Nishimura S Manabe I Hashimoto H Ohnishi Y Ogata H Tokuyama K Tsunoda M Ide T Murakami K Nagai R Kadowaki T Overexpression of monocyte chemoattractant protein-1 in adipose tissues causes macrophage recruitment and insulin resistance J Biol Chem 2006 281 26602 26614 10.1074/jbc.M601284200 16809344

[B34] Brodmerkel CM Huber R Covington M Diamond S Hall L Collins R Leffet L Gallagher K Feldman P Collier P Stow M Gu X Baribaud F Shin N Thomas B Burn T Hollis G Yeleswaram S Solomon K Friedman S Wang A Xue CB Newton RC Scherle P Vaddi K Discovery and pharmacological characterization of a novel rodent-active CCR2 antagonist, INCB3344 J Immunol 2005 175 5370 5378 16210643 10.4049/jimmunol.175.8.5370

[B35] Ogilvie P Paoletti S Clark-Lewis I Uguccioni M Eotaxin-3 is a natural antagonist for CCR2 and exerts a repulsive effect on human monocytes Blood 2003 102 789 794 10.1182/blood-2002-09-2773 12689946

[B36] Baggiolini M Chemotactic and inflammatory cytokines - CXC and CC proteins Adv Exp Med Biol 1993 351 1 11 7942288 10.1007/978-1-4615-2952-1_1

[B37] de Lemos JA Morrow DA Sabatine MS Murphy SA Gibson CM Antman EM McCabe CH Cannon CP Braunwald E Association between plasma levels of monocyte chemoattractant protein-1 and long-term clinical outcomes in patients with acute coronary syndromes Circulation 2003 107 690 695 10.1161/01.CIR.0000049742.68848.99 12578870

[B38] Coll B van Wijk JP Parra S Castro Cabezas M Hoepelman IM Alonso-Villaverde C de Koning EJ Camps J Ferre N Rabelink TJ Tous M Joven J Effects of rosiglitazone and metformin on postprandial paraoxonase-1 and monocyte chemoattractant protein-1 in human immunodeficiency virus-infected patients with lipodystrophy Eur J Pharmacol 2006 544 104 110 10.1016/j.ejphar.2006.06.014 16843455

[B39] Christiansen T Richelsen B Bruun JM Monocyte chemoattractant protein-1 is produced in isolated adipocytes, associated with adiposity and reduced after weight loss in morbid obese subjects Int J Obes (Lond) 2005 29 146 150 10.1038/sj.ijo.0802839 15520826

[B40] Chung S Lapoint K Martinez K Kennedy A Boysen Sandberg M McIntosh MK Preadipocytes mediate lipopolysaccharide-induced inflammation and insulin resistance in primary cultures of newly differentiated human adipocytes Endocrinology 2006 147 5340 5351 10.1210/en.2006-0536 16873530

[B41] Kratchmarova I Kalume DE Blagoev B Scherer PE Podtelejnikov AV Molina H Bickel PE Andersen JS Fernandez MM Bunkenborg J Roepstorff P Kristiansen K Lodish HF Mann M Pandey A A proteomic approach for identification of secreted proteins during the differentiation of 3T3-L1 preadipocytes to adipocytes Mol Cell Proteomics 2002 1 213 222 10.1074/mcp.M200006-MCP200 12096121

[B42] Lumeng CN Bodzin JL Saltiel AR Obesity induces a phenotypic switch in adipose tissue macrophage polarization J Clin Invest 2007 117 175 184 10.1172/JCI29881 17200717 PMC1716210

[B43] Haase H Ober-Blobaum JL Engelhardt G Hebel S Heit A Heine H Rink L Zinc signals are essential for lipopolysaccharide-induced signal transduction in monocytes J Immunol 2008 181 6491 6502 18941240 10.4049/jimmunol.181.9.6491

[B44] Mendez-Samperio P Palma J Vazquez A Roles of intracellular calcium and NF-kappaB in the bacillus Calmette-Guerin-induced secretion of interleukin-8 from human monocytes Cell Immunol 2001 211 113 122 10.1006/cimm.2001.1816 11591115

[B45] Sarau HM Rush JA Foley JJ Brawner ME Schmidt DB White JR Barnette MS Characterization of functional chemokine receptors (CCR1 and CCR2) on EoL-3 cells: a model system to examine the role of chemokines in cell function J Pharmacol Exp Ther 1997 283 411 418 9336350

